# Normal saline-induced deoxygenation of red blood cells probed by optical tweezers combined with the micro-Raman technique

**DOI:** 10.1039/c8ra10061f

**Published:** 2019-03-11

**Authors:** Jijo Lukose, Mithun N, Ganesh Mohan, Shamee Shastry, Santhosh Chidangil

**Affiliations:** Centre of Excellence for Biophotonics, Department of Atomic and Molecular Physics, Manipal Academy of Higher Education Manipal Karnataka India-576104 santhosh.cls@manipal.edu; Department of Immunohematology and Blood Transfusion, Kasturba Medical College, Manipal Academy of Higher Education Manipal Karnataka India-576104

## Abstract

The use of normal saline for washing red blood cells and treating critically ill patients is a regular medical practice in hospital settings. An optical tweezer in combination with Raman spectroscopy is an analytical tool employed for the investigation of single cell dynamics, thus providing molecular fingerprint of the cell by optically trapping the cell at a laser focus. In this study, the impact of normal saline on individual human red blood cell was compared with that of blood plasma using Raman tweezers spectroscopy. Major spectral variations in the marker frequencies at 1209 cm^−1^, 1222 cm^−1^, 1544 cm^−1^, and 1561 cm^−1^ of the Raman spectrum of the treated cells imply that the transition of hemoglobin to the deoxygenated state occurs when 0.9% normal saline is used. This may result in serious implications in blood transfusion. The results obtained from the principal component analysis also displayed clear differentiation among the red blood cells diluted in normal saline and those diluted in plasma. In future studies, efforts will be made to correlate the deoxygenation status of red blood cells with various human disorders.

## Abstract 2

Micro-Raman spectroscopy of optically trapped live red blood cell demonstrates normal saline induced deoxygenation.

## Graphical abstract

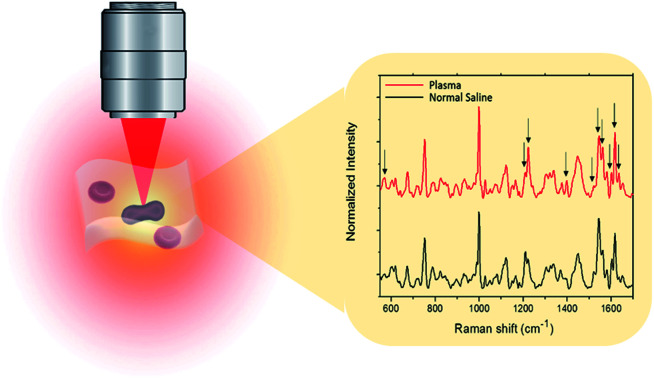

## Introduction

The benefit *versus* safety of normal saline in the medical field is a subject of debate; in this regard, many researchers have reviewed the safety of using normal saline (0.9% NaCl) in different clinical settings^1^ since it is one of the most commonly used solutions in the hospital settings. In transfusion medicine, normal saline is used for cell washing and salvaging, in apheresis and for the resuscitation of patients with blood or fluid loss. Washing of red blood cells (RBCs) is carried out using 1 or 2 liters of sterile normal saline. This process is typically performed to remove plasma proteins and glycerol from the frozen RBC units. As the process of washing removes the anticoagulants and preservative solution, the shelf life of the RBC product is reduced. As per the American Association of Blood Banking guidelines, the washed RBC unit can be stored at 1–6 °C for 24 hours.^2^ During apheresis, normal saline is used to prime the circuit and as a replacement fluid in the therapeutic plasma exchange procedures. Moreover, it is used during intra-operative cell salvaging to wash the red blood cells. In a recent study, normal saline could induce higher levels of hemolysis as compared to Plasma-Lyte A after the cells were washed with it and stored for a short term (24 h or less).^3^ Previously, low levels of hemolysis were not a matter of concern as they were thought to be harmless and frequently present in transfused red cells; however, they may be injurious and need to be further researched; this observation is very critical considering that even low levels of hemolysis can trigger the probability of vital organ failure, vasculopathy, and predispose to nosocomial infection. Infusion of normal saline in high volumes during resuscitation and apheresis can cause hyperchloremic metabolic acidosis, which may impair the renal function.^4^ Normal saline is also commonly used as an isotonic buffer for red blood cells in research protocols. Some groups have also used it for spectroscopic investigation of live red blood cells.^5^

The emergence and major developments occurring in the interdisciplinary “biophotonics” area have contributed to the origin of many spectroscopic techniques specialized in the examination of a plethora of biological/biochemical processes. The knowledge of various blood components, such as blood cells, platelets and white blood cells, is of paramount importance in recognizing different conditions of the human body. Raman spectroscopy is widely regarded as a highly reliable optical tool for investigating biological systems.^6,7^ However, any type of direct analysis of live cells suspended in a liquid medium is highly restricted by the random movement of these micron-sized particles due to Brownian motion. This problem can be tackled using optical tweezers, which capture a microscopic particle with the use of a tightly focused laser beam. Optical tweezers, developed by Arthur Ashkin, have been gaining significant attention from the scientific fraternity, especially from biophysicists, due to their ability to manipulate and trap a single cell.^8,9^ The use of optical tweezers in combination with Raman spectroscopy has opened up new avenues for the inspection of live cells and their interactions.^10^ Basically, in the Raman tweezers system, a beam is used to trap a single live cell, and the same or another laser beam simultaneously excites and generates Raman signals. Thus, the cell can be easily manipulated for the required studies. The advantages of the Raman tweezers technique are its high specificity to identify the chemical moieties of an individual biological cell and real-time identification of the biochemical alterations in the presence of external stimuli; moreover, minimal sample preparation and sample volume are required in this technique. In addition, since it is a label-free technique, the time-consuming fluorescent or radioactive labelling of the biological analytes is not required. Researchers have already applied this tool to obtain vital information regarding the biochemical alterations occurring in a single, live red blood cell due to external factors such as temperature, laser irradiation, silver nanoparticles, glucose *etc.*^11–14^ Raman tweezers technique was also explored for monitoring the oxygenation states of hemoglobin in red blood cells and their relation with various human health disorders.^15,16^ This technique has several applications in medicine as well as in cell biology, diagnosing cell disorders by investigating the cellular mechanisms at the individual cell level.^17–19^

The comparison of normal saline with blood plasma as a medium for red blood cell studies is an important area of research; however, the use of Raman tweezers to probe normal saline-induced modifications on a single RBC has not been reported to date. To the best of our knowledge, this is the first study describing the saline-induced spectral changes of red blood cells. This study presents the micro-Raman spectroscopic comparison of a single, live red blood cell optically trapped and suspended in two media: normal saline and human blood plasma. Significant alterations in the known oxygenation marker bands reveal a transition from the oxy to the deoxy state when red blood cells are suspended in normal saline.

## Experimental

### Sample preparation

Sample collection was conducted by the Blood Bank, Kasturba Medical College, Manipal, India. The national guidelines on blood donor acceptance were followed to select the study subjects.^20^ Informed consent was obtained from all subjects. All the donors in the study were males, and their age was between 18 and 60 years. All the samples had a hemoglobin level above 12.5 g dl^−1^. Moreover, infection screening tests for HIV, HBV, HCV, malaria and syphilis were found to be negative for the samples. Fresh whole blood obtained from the volunteers was centrifuged for 5 minutes at 3000 rpm to separate the packed red blood cells (PRBC). The study subjects did not consume any ethanol-containing products for 24 hours prior to sampling; in this procedure, the packed red blood cells obtained from the volunteers were highly diluted and suspended in blood plasma or a normal saline solution for ten minutes prior to conducting the Raman measurements. AB blood group plasma obtained from the volunteers selected based on the national guidelines was used for the study.^20^ The dilution was made to avoid multiple cell trapping due to laser spot. All Raman spectra were acquired within two hours after the blood was obtained and diluted in the media solution. The power levels that are used to obtain the Raman spectra of the treated red blood cells in both the saline and the plasma are well below the threshold for the occurrence of photo-dissociation. In all the studies, measurement was performed using normal saline (0.9% NaCl obtained from Infutec Healthcare Limited), and the PRBC suspension in blood plasma was used as a control. Permission was obtained from the Institutional Ethics Committee, Kasturba Medical College and Kasturba Hospital, on 17^th^ January 2018 (IEC: 68/2018) before initiation of the study.

### Instrumentation

**Fig. 1:**
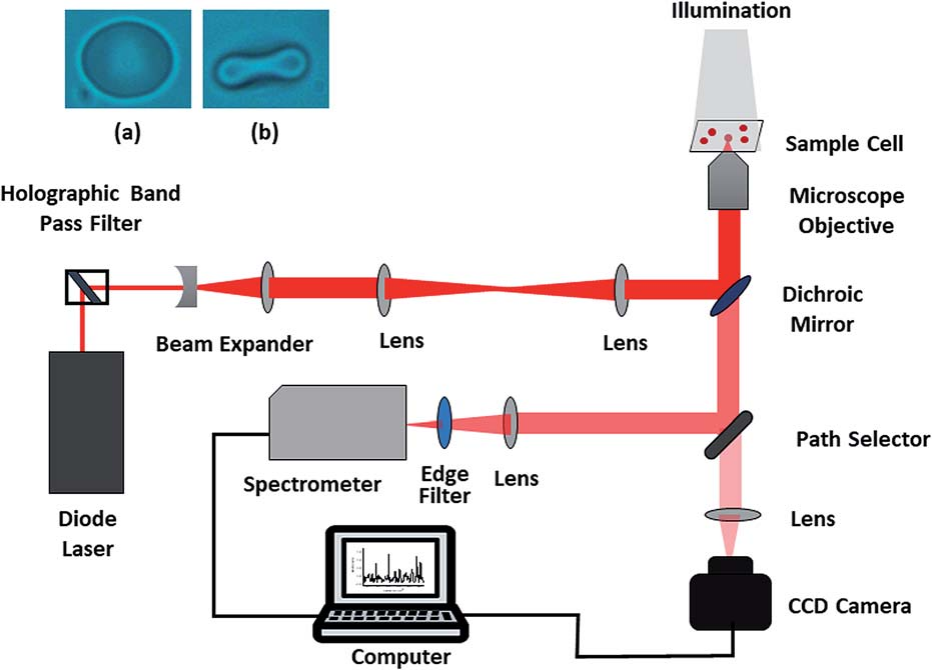
Schematic of the Raman tweezers experimental setup; the inset shows RBC (a) before and (b) after trapping.

The present study was carried out using a custom-built, single-beam Raman tweezers instrument (Fig. 1).^21^ A 785 nm laser beam (Starbright Diode Laser, Torsana Laser Tech, Denmark) was used for trapping as well as excitation of the live red blood cells diluted in isotonic media (0.9% NaCl medium or Plasma). An inverted microscope (Nikon Eclipse, Ti-U, Japan) with a high numerical aperture (1.3 NA) and 100X oil immersion microscope objective (Nikon, Plan Fluor, Japan) was used to realize the tight focusing of the laser beam. The resultant scattered Raman signals were obtained using the same objective and directed towards a spectrometer (Horiba Jobin Yvon iHR320 with 1200 grooves per mm grating blazed at 750 nm) and liquid nitrogen-cooled CCD (Symphony CCD-1024 × 256-OPEN-1LS). Since the laser exposure time on an individual cell was restricted to 60 s and a low power of ∼7.5 mW was used, the chance of photo damage to the cell was little; thus, the cell remained intact, and the resultant Raman spectrum was valid. The spectra were never obtained on the same red blood cell repeatedly to minimize the photo-damage. Baseline correction as well as normalization of the Raman spectra after smoothing were performed using MATLAB *via* the vector normalization method. The image of RBC obtained before and after trapping is shown in the inset of Fig. 1.

## Results and discussion

**Fig. 2:**
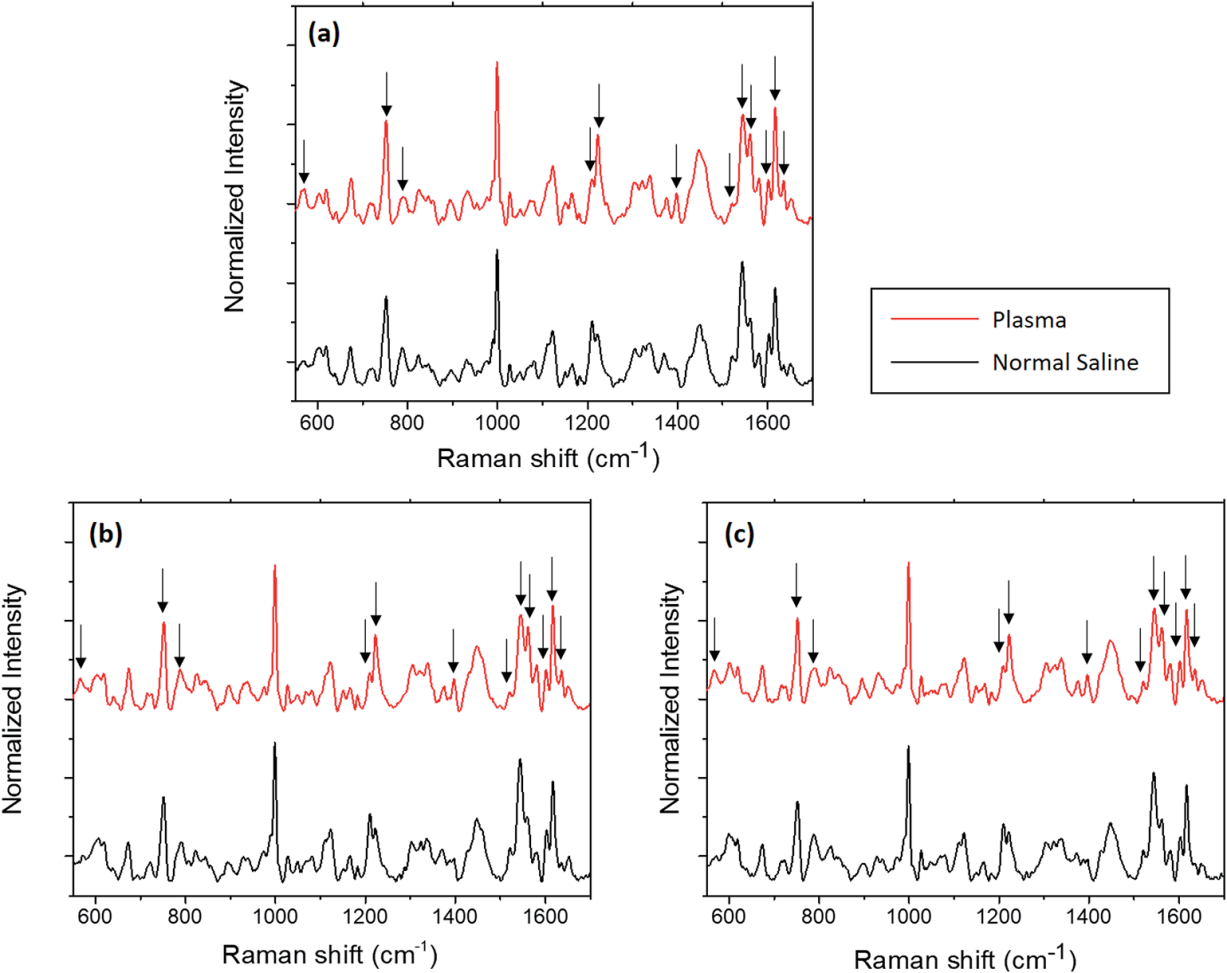
Average Raman spectra of RBC diluted in normal saline and plasma from three volunteers (a)–(c).

In this section, the changes observed for the optically trapped single RBC when the suspended medium is changed from plasma to normal saline have been discussed using Raman spectroscopy. The comparative spectra of the RBC diluted in normal saline and blood plasma are shown in Fig. 2, in which the major spectral variations are highlighted. The acquisition time for each spectrum was 60 s with two accumulations. Each spectrum shown in the figure is an average of the spectra of 5 different cells obtained from the same individual. To validate the spectral changes, the experiment was repeated by obtaining the blood from two more volunteers, and the corresponding spectra are shown in Fig. 2(b) and (c). The experiments for each set of samples were performed in less than one hour.

Hemoglobin is the major molecular species in the cytoplasm of RBCs, and the majority of peaks present in the Raman spectra of the red blood cell originate from hemoglobin. Hemoglobin is responsible for the transport of oxygen *via* binding of the heme groups to oxygen. In general, this protein is described by a two-state model between two alternate structures: (1) a deoxygenated (deoxy), tense (T) structure and (2) an oxygenated (oxy), relaxed (R) structure. The form of hemoglobin that is bound to oxygen is termed as oxyhemoglobin, whereas the form that is not bound to oxygen is termed as deoxyhemoglobin.^22^ A hemoglobin molecule consists of four polypeptide globin chain subunits that comprise a heme group in each of it. This heme group is composed of a porphyrin ring that has an iron atom in its centre with six coordination sites; the four coordination sites of iron are occupied by the porphyrin nitrogen, whereas the fifth site is connected to the histidine residue in the globin chain.^23^ Once the sixth coordination site of iron binds with oxygen, the iron atom lies in the porphyrin plane and thus adopts a planar configuration in the oxygenated state.^24^ Once oxygen is removed from the heme, the iron atom is pulled out of the porphyrin plane towards the histidine residue; this results in a domed configuration for the deoxygenated form.^24,25^ The peaks displaying the major changes induced by normal saline are presented in Table 1 along with their band assignments.^21^

**Table 1:** Band assignments of RBCs that underwent major changes in the presence of normal saline

Wavenumber (cm^−1^)	Intensity variation	Band assignment
565	↓	*ν*(Fe–O_2_)
752	↓	*ν*_15_
1209	↑	*ν*_5_ + *ν*_18_
1222	↓	*ν*_13_ or *ν*_42_
1397	↓	*ν*_20_
1521	↑	*ν*_38_
1544	↑	*ν*_11_
1561	↓	*ν*_2_
1603	↑	*ν*(C=C)_vinyl_
1617	↓	*ν*(C=C)_vinyl_
1636	↓	*ν*_10_

**Fig. 3:**
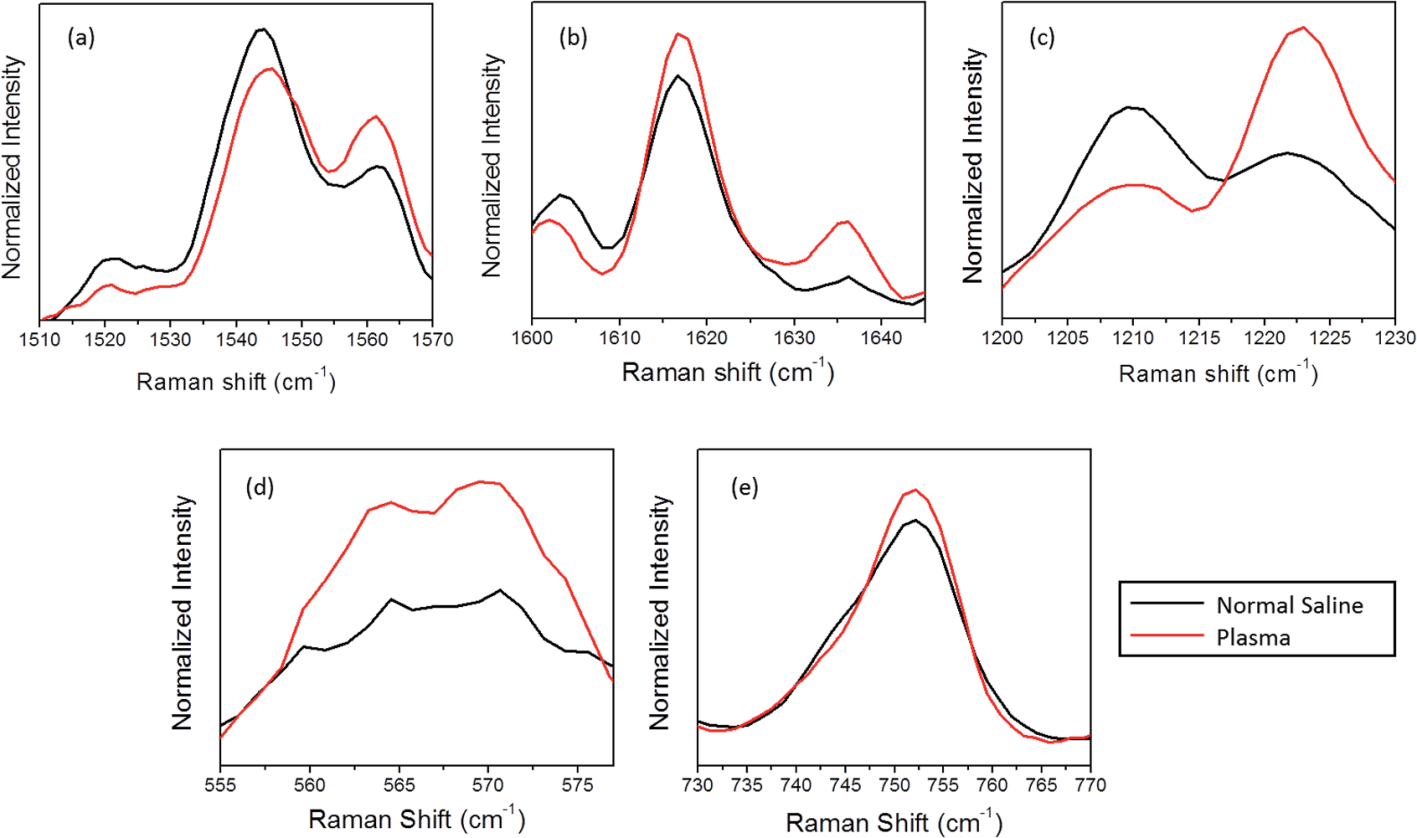
Average Raman spectra of RBCs diluted in normal saline and plasma where spectral variations are dominant (a), (b) the spin marker region, (c) the methine deformation region, (d) the FeO_2_ stretch, and (e) the porphyrin breathing mode.

Primarily, the oxygenation trend is verified *via* inspecting the spin-state marker region of heme, as shown in Fig. 3(a) and (b). The spin-state marker region (1500–1650 cm^−1^) is regarded as an important region of interest due to the presence of prominent Raman bands related to the oxygenation state of hemoglobin. The Raman signals appearing at 1544 cm^−1^, 1561 cm^−1^, 1617 cm^−1^, and 1636 cm^−1^ are all contributed by the C–C bonds in the porphyrin ring, which rely on the spin states of the iron atom.^26^ As abovementioned, during the transition from the oxy to the deoxy state, the iron atom in the ferric low spin state is converted to the ferrous form due to electron withdrawal; this forces the displacement of the Fe atom 4 nm out of the porphyrin plane.^25,27^ This conformational change will be immediately reflected in the vibrational modes linked with the porphyrin ring, which can be tracked *via* the Raman spectra. The increase in the intensity of 1521 cm^−1^, 1544 cm^−1^ as well as 1603 cm^−1^ accompanied by a decrease in the intensity of 1561 cm^−1^ can be assigned to the presence of more number of deoxy hemes in the blood cell. In the case of normal saline, the peaks present at 1617 cm^−1^ band and 1636 cm^−1^ (*δ*(CɑCm)asym) band experienced a reduction in intensity; this could be linked with porphyrin doming.^26^

Furthermore, the oxygenation trends in the red blood cells diluted with saline and plasma were revaluated by monitoring the bands present in the 1200–1230 cm^−1^ spectral region (Fig. 3(c)). These are the spectral regions assigned to the methine C–H deformations in the heme, which are readily influenced by the coordination between the porphyrin ring and the iron atom.^28^ Any conformational change that occurs in hemoglobin leads to an immediate variation in the deformation angle of the C–H vibrations due to the proximity of these vibrations to the protein subunits.^24^ This subsequently results in the Raman shift of the corresponding bands due to the transition from the oxygenated to the deoxygenated state. In the case of normal saline, a gain in the intensity of 1209 cm^−1^ peak accompanied by a loss in the intensity of 1222 cm^−1^ peak was observed. Earlier studies have correlated this intensity shift with the oxy-deoxy state transition due to the conformational changes occurring in heme.^29^ To confirm the saline-induced deoxygenation, the well-known spectral region corresponding to heme-bound oxygen and the FeO_2_ stretch present at 565 cm^−1^ (Fig. 3(d)) are considered. In the case of normal saline, the drop in the O_2_ concentration is clearly visible from the decline in the intensity of the band at 565 cm^−1^.

The overlaid plot of the spectral region centered at 752 cm^−1^ is shown in Fig. 3(e). This peak corresponds to the C–N–C vibration in the porphyrin ring, which is generally used as an indicator for the number of intact hemoglobin molecules present in red blood cells.^5^ In general, the intensity of the porphyrin breathing mode is regarded as a marker for the vitality of RBCs. A decrease in the intensity of the band is observed for normal saline as compared to the case of blood plasma. This variation also questions the stability of RBCs in normal saline for hospital applications. Similar to the spin-state marker region, the pyrrole ring stretching region (1300–1400 cm^−1^) is highly sensitive to the oxidation state of iron present in the porphyrin ring. A decrease in the intensity of the band at 1397 cm^−1^, which represents the pyrrole quarter ring stretching, is found in the case of the normal saline samples (Fig. 2(a)). In addition, the band assigned to the pyrrole half ring stretching, which appears at 1375 cm^−1^ in the oxygenated state, is shifted to 1370 cm^−1^ in the case of normal saline. The presence of the 787 cm^−1^ peak observed for the RBC diluted with normal saline also ascertains the abovementioned fact.^29^ This again corroborates with the assumption of the deoxygenated state transition in red blood cells in the presence of saline.

**Fig. 4:**
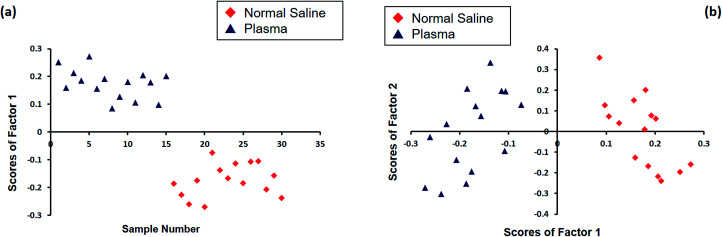
The PCA analysis plot of Raman spectra of the RBCs diluted in normal saline and blood plasma: (a) sample number *vs.* scores of factor 1 and (b) scores of factor 1 *vs.* scores of factor 2.

Principal component analysis (PCA) was performed using the GRAMS software to obtain statistical discrimination among the spectral information of RBCs suspended in normal saline and plasma. This statistical analysis was carried out on a total of 30 spectra, with 15 spectra each for the two sets of cells separately diluted in plasma and in saline obtained from three different individuals. Fig. 4 shows the PCA results obtained for normal saline and plasma in the region 1100–1700 cm^−1^, where the major oxygenation markers are observed. Fig. 4(a) represents the plot between the sample number and the score of factor 1. It is evident from the plot that all the normal saline samples have negative scores, and the plasma-incubated samples have positive scores. The plot between the scores of factor one and the scores of factor two is depicted in Fig. 4(b). A clear distinction was observed between the Raman spectra of the two classes: red blood cells in normal saline as one class and plasma-diluted samples as the other. Most of the RBCs in normal saline lie in the first and second quadrants, whereas all the plasma samples appeared in the third and fourth quadrants.

As is well-known, more than 90% of hemoglobin is oxygenated during the time of blood donation.^30^ However, in the present case, the bands representing the deoxygenation markers displayed a remarkable intensity enhancement for normal saline as compared to the case of blood plasma. This observation is drawn by monitoring the marker peaks mainly at 1209 cm^−1^, 1521 cm^−1^, 1544 cm^−1^ and 1602 cm^−1^. Thus, we observed strong evidence for the increased deoxygenation of hemoglobin of the red blood cells diluted with normal saline. The results obtained are in good agreement with those reported in previous studies regarding the oxygenation states of hemoglobin.^29,31^ Studies have already been reported on the deoxygenation of red blood cells *via* laser-induced optical trapping, mechanical stretching, *etc.*^26,31^ Although Raman tweezers are widely used to study the oxygenation status of red blood cells, normal saline-induced deoxygenation has not been reported to date. This is the first report describing the normal saline-induced deoxygenation in RBCs investigated using the Raman tweezers technique. Even in the recent studies on the Raman tweezers investigation of red blood cells, normal saline has been utilized as media, which results in obtaining deoxygenated spectra for control RBCs.^32^ This study also highlights the necessity of performing investigations on the red blood cells in plasma to track the eventual deoxygenation of hemoglobin.

**Fig. 5:**
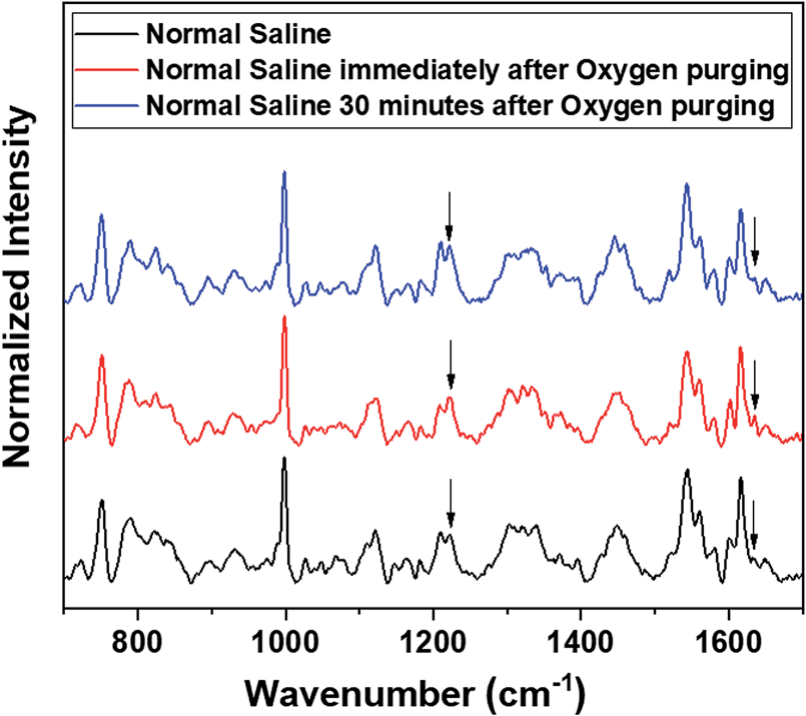
Oxygen purging of RBC in normal saline.

**Fig. 6:**
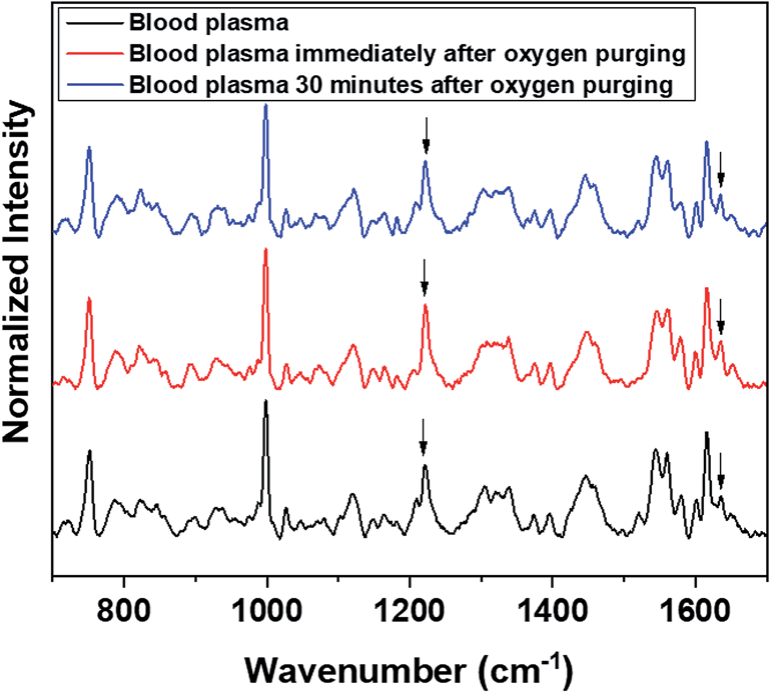
Oxygen purging of RBC in plasma.

Raman measurements were also performed by saturating the sample solutions (RBCs in normal saline as well as in blood plasma) *via* purging medical oxygen for one hour. The experimental results have again ascertained the hemoglobin deoxygenation tendency of red blood cells in normal saline. Even after oxygen purging, the spectra remained the same as those of the deoxygenated hemoglobin in the case of RBCs suspended in normal saline. Although the initial spectra obtained immediately after oxygen purging (Fig. 5) indicated a slight increase in the oxygenation tendency, this instant trend was found to be unstable, and it did not sustain in normal saline for more time. It is clearly evident from the switching back of the ratio 1209 cm^−1^/1222 cm^−1^ (peaks in the methine deformation region) and the reversal in the intensity of the oxygenation marker at 1639 cm^−1^ (Fig. 5) that saline induces and maintains the deoxygenation trend in red blood cells. In the case of blood plasma, oxygenation increases during oxygen purging; this can be clearly observed from the methine deformation region shown in Fig. 6. The oxygenation trend is stable and maintained in the case of blood plasma.

**Fig. 7:**
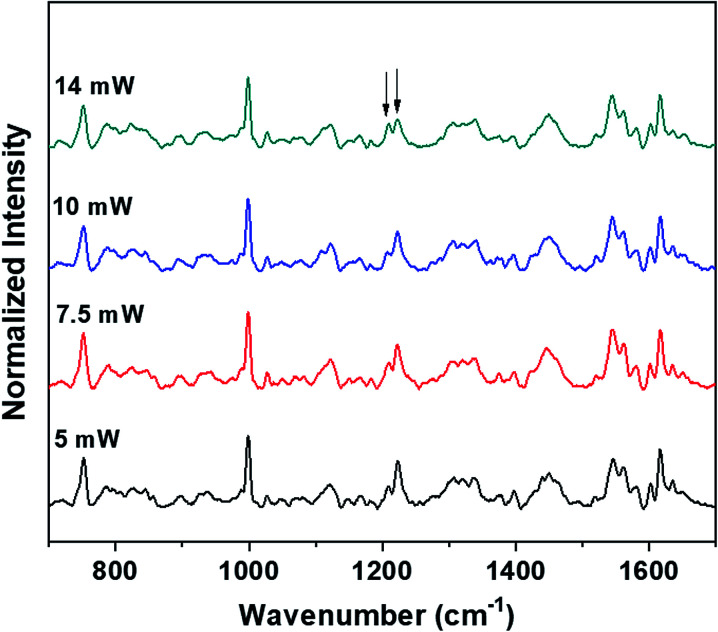
Power-dependent Raman spectra of RBC in blood plasma.

The power levels that are used to obtain the Raman spectra of the control (blood plasma) and normal saline-incubated red blood cells are well below the threshold for the occurrence of laser-induced deoxygenation of RBCs. The dependence of power on the hemoglobin oxygenation states for blood plasma was investigated. As shown in Fig. 7, spectra were obtained with different laser powers for the optically trapped RBCs suspended in blood plasma. It is clear from the Raman spectra that significant changes in the hemoglobin oxygenation states are observed only after 14 mW. There were no obvious changes till the laser power reached 10 mW. Thus, it is clearly evident from the results that the variations, including intensity flipping of the methine deformation peaks at 1209 cm^−1^ and 1222 cm^−1^, are not photo-induced due to the presence of the 785 nm laser beam. Hence, the spectral changes presented in Fig. 2 and 3 are entirely ascribable to the saline-induced changes in the heme conformations.

Transfusion of the packed red cell concentrate (PRBC) to critically ill patients plays a vital role in maintaining the oxygen saturation levels of tissues, thereby preventing hypoxia-induced organ damage. Most of the times, patients in the intensive care units (ICU) have an increased demand of PRBC transfusions because of their clinical conditions and low hemoglobin levels. These patients will also be receiving normal saline as intra venous fluid therapy to support their intravascular volume status. Apart from the use of normal saline as IV fluids, washing of red cells is conducted with normal saline, which can alter the PRBCs' oxygen-carrying capacity. The *in vivo* effect of normal saline on the oxygen-carrying capacity has to be considered because the current study clearly shows that the hemoglobin molecule changes its conformation when it is mixed with normal saline *ex vivo*.

## Conclusions

Herein, Raman tweezers spectroscopy was explored to investigate the oxygenation state transitions of red blood cells in plasma and 0.9% normal saline. This, in turn, could be detected by monitoring the wavenumber shifts associated with the Raman marker bands for the R (relaxed) to the T (tensed) transitions. From the spectral comparison and assessment, the study concluded the transformation of hemoglobin from the oxy state to deoxy state when the cell medium was changed from blood plasma to normal saline. In addition, the principal component analysis supported the spectral variations obtained *via* the Raman studies by displaying significant differentiation among the red blood cells suspended in normal saline and those suspended in blood plasma. The present results demand more investigations on the evaluation of the impact of saline-induced deoxygenation on red blood cells, especially in medical settings.

## Conflicts of interest

There are no conflicts to declare.

## Supplementary Material
